# CDROM: Classification of Duplicate gene RetentiOn Mechanisms

**DOI:** 10.1186/s12862-016-0644-x

**Published:** 2016-04-14

**Authors:** Brent R. Perry, Raquel Assis

**Affiliations:** Department of Biology, Pennsylvania State University, University Park, PA 16802 USA

**Keywords:** Gene duplication, Neofunctionalization, Subfunctionalization, Gene expression evolution

## Abstract

**Background:**

Gene duplication is a major source of new genes that is thought to play an important role in phenotypic innovation. Though several mechanisms have been hypothesized to drive the functional evolution and long-term retention of duplicate genes, there are currently no software tools for assessing their genome-wide contributions. Thus, the evolutionary mechanisms by which duplicate genes acquire novel functions remain unclear in a number of taxa.

**Results:**

In a recent study, researchers developed a phylogenetic approach that uses gene expression data from two species to classify the mechanisms underlying the retention of duplicate genes (Proc Natl Acad Sci USA 110:1740917414, 2013). We have implemented their classification method, as well as a more generalized method, in the R package *CDROM*, enabling users to apply these methods to their data and gain insights into the origin of novel biological functions after gene duplication. The *CDROM* R package, source code, and user manual for the R package are available for download from CRAN at https://cran.rstudio.com/web/packages/CDROM/. Additionally, the *CDROM* R source code, user manual for running *CDROM* from the source code, and sample dataset used in this manuscript can be accessed at www.personal.psu.edu/rua15/software.html.

**Conclusions:**

*CDROM* is the first software package that enables genome-wide classification of the mechanisms driving the long-term retention of duplicate genes. It is user-friendly and flexible, providing researchers with a tool for studying the functional evolution of duplicate genes in a variety of taxa.

## Background

Gene duplication produces two copies of an existing gene—one that arose from the same common ancestor (parent), and a new copy that is the product of the duplication event (child). Long-term retention of a pair of duplicate genes can occur via preservation of ancestral functions in both copies (conservation; [9]), preservation of ancestral functions in one copy and acquisition of a new function in the other (neofunctionalization; [9]), division of ancestral functions between copies (subfunctionalization; [4, 6, 12]), or acquisition of new functions in both copies (specialization; [5]). Knowledge of the genome-wide contributions of these evolutionary mechanisms can provide insight into the emergence of complex phenotypes after gene duplication.

Assis and Bachtrog [2] recently developed a phylogenetic approach that classifies the mechanisms retaining duplicate genes by comparing spatial gene expression profiles of duplicate genes in one species to those of their ancestral genes in a second species. For each pair of duplicates, they compared expression profiles among a triplet of genes—the parent copy (P), the child copy (C), and the ancestral gene in a sister species (A). They calculated Euclidian distances between expression profiles of each duplicate gene and the ancestral gene (*E*_P,A_ and *E*_C,A_), as well as between the combined parent-child gene expression profile and the ancestral gene expression profile (*E*_P + C,A_). They also calculated Euclidian distances between expression profiles of orthologous genes (those that arose from the same common ancestor) present in a single copy in both sister species (*E*_S1,S2_), which they used to establish a cutoff for expression divergence (denoted as *E*_div_ here). Then, they classified the four retention mechanisms by applying the following phylogenetic rules: conservation if *E*_P,A_ ≤ *E*_div_ and *E*_C,A_ ≤ *E*_div_; neofunctionalization if *E*_P,A_ > *E*_div_ and *E*_C,A_ ≤ *E*_div_, or if *E*_P,A_ ≤ *E*_div_ and *E*_C,A_ > *E*_div_; subfunctionalization if *E*_P,A_ > *E*_div_, *E*_C,A_ > *E*_div_, and *E*_P + C,A_ ≤ *E*_div_; or specialization if *E*_P,A_ > *E*_div_, *E*_C,A_ > *E*_div_, and *E*_P + C,A_ > *E*_div_ [2].

## Implementation

Here, we present *CDROM*, an R package that implements Assis and Bachtrog’s [2] phylogenetic classification method. To run *CDROM*, the user provides a table of duplicate genes and their ancestral genes in a sister species, a table of single-copy orthologous genes, and tables containing gene expression data for both species. Gene expression data can be for a single sample (*n* = 1) or for multiple samples (*n* > 1), e.g., from different cells or tissues (as used in [2, 3]), developmental time points, or experimental conditions. The number of samples determines the number of dimensions in which Euclidian distances are calculated. Thus, *CDROM* can even be used when there is a single expression data point from a single-celled organism. It should be noted that it is possible to apply *CDROM* to data for any quantitative trait. However, because the method was only tested on gene expression data, users should demonstrate caution when analyzing results and making inferences from other types of data.

*CDROM* first obtains expression profiles for all genes by converting raw expression levels to relative expression values (proportions of contribution to total gene expression). Next, it computes Euclidian distances from gene expression profiles. Then, it uses the phylogenetic rules defined by Assis and Bachtrog [2] to classify the retention mechanism of each duplicate gene pair. In the classification step, the semi-interquartile range (SIQR) from the median of the *E*_S1,S2_ distribution is set as the default *E*_div_ because of its robustness to distribution shape and outliers. However, the user also has the option to specify *E*_div_. To aid the user in selecting *E*_div_, *CDROM* provides counts of classifications obtained with five *E*_div_ values. Thus, the user can choose *E*_div_ by comparing results obtained with different values, and also explore the sensitivity of classifications to *E*_div_, as was done in previous studies [2, 3].

It is important to note that, while *CDROM* performs classification of duplicate gene retention mechanisms, it does not identify duplicate, single-copy, and orthologous genes or distinguish between parent and child duplicate gene copies. *CDROM* does not include these features because the types, availability, and quality of genome sequence, annotation, and alignment data vary across taxa, making it difficult to automate these analyses so that they are broadly applicable. Moreover, there are several sophisticated software tools for identifying duplicate genes and orthologs (e.g., [1, 7, 8, 11]), and sometimes these data are available from publicly available databases (e.g., [10, 13]). While there are currently no automated approaches for distinguishing between parent and child duplicate gene copies, this analysis requires knowledge about both gene sequences and their genomic positions (synteny), and is thus largely dependent on data availability and quality. Because appropriate data for this analysis are often unavailable, and because it can sometimes be difficult or impossible to distinguish between parent and child copies even with appropriate data, *CDROM* defaults to a generalized version of Assis and Bachtrog’s [2] method that does not require parent/child specification. With the default method, the user is still able to address important evolutionary questions about the mechanisms retaining duplicate genes. Thus, knowledge of parent-child relationships is not necessary, and only enables refinement of the answers to these questions.

A limitation of Assis and Bachtrog’s [2] approach, and consequently of our software, is that gene expression only represents one facet of gene function. In particular, there may be more power to detect functional divergence if our software utilized additional sources of information, such as gene sequences or protein-protein interaction data. However, there are several reasons why we did not allow for multiple types of data as input to *CDROM*. First, it is unclear how to combine different types of data without fundamentally changing the approach described by Assis and Bachtrog [2]. Second, there is the possibility of disagreement among different types of data, making the classification problem much more complex. Finally, researchers may not have access to more than one type of data, which would limit the scope of our software to those who do. However, a major strength of *CDROM* is that it runs quickly. Thus, our suggestion to researchers with multiple datasets is to run *CDROM* separately on each dataset, and then compare the results obtained for different types of data. A possible avenue for future improvement of *CDROM* is to combine information from multiple types of data and include this functionality as a user-defined option, thereby still enabling those with only one type of data to use our software.

## Results and discussion

**Fig. 1:**
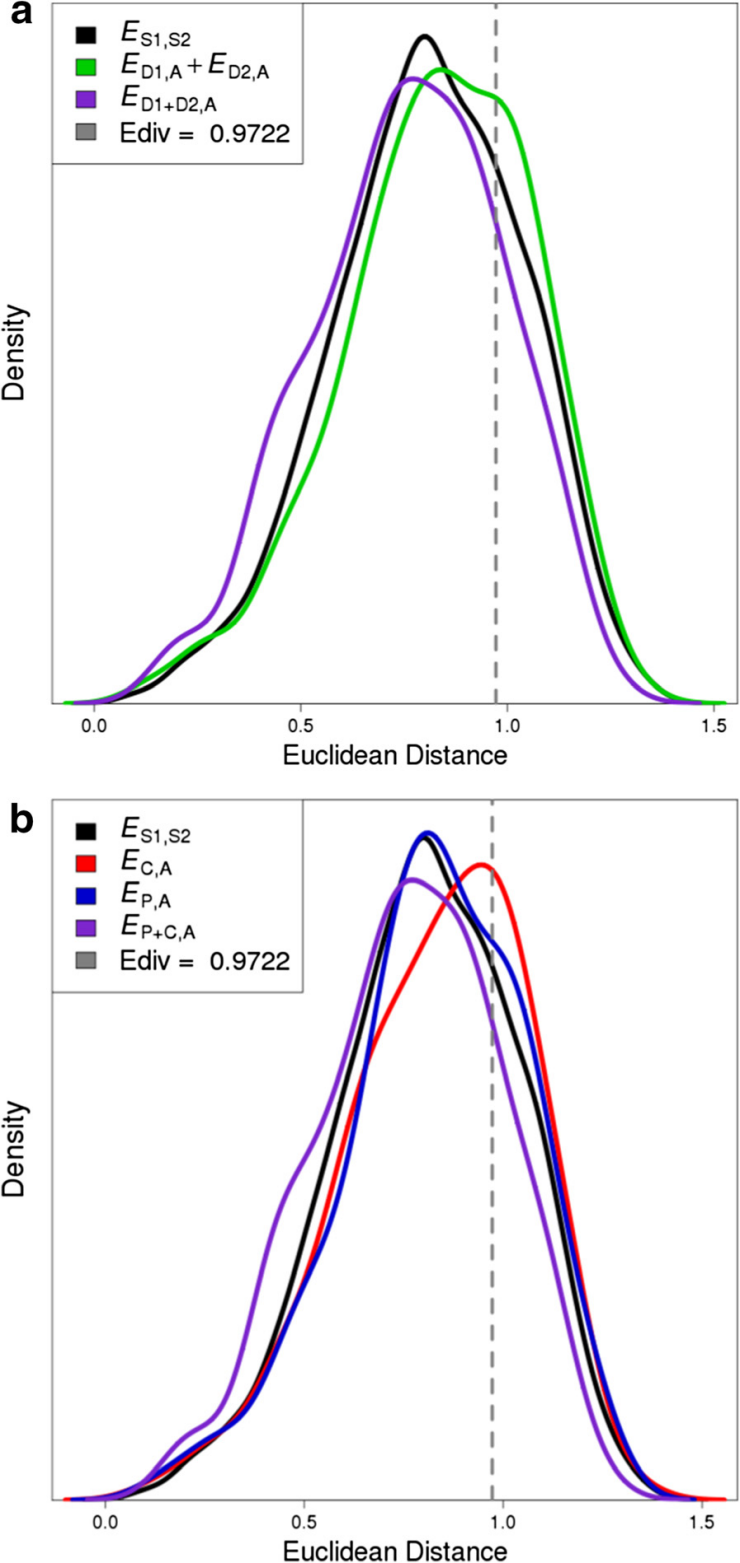
Examples of the figure output by *CDROM. CDROM* outputs a figure showing distributions of all computed distances and the position of *E*_div_. Here, *CDROM* was applied to spatial gene expression data from duplicate genes that arose after human-chicken divergence (from [3]). Under the default method (**a**), a single distribution is plotted for *E*_D1,A_ and *E*_D2,A_ (indicated by *E*_D1,A_ + *E*_D2,A_). When parent and child copies are specified (**b**), separate distributions are plotted for *E*_P,A_ and *E*_C,A_

*CDROM* outputs one figure and two tables. The figure shows distributions of the distances calculated and the position of the chosen *E*_div_ (either default or user-specified), the first table indicates the classification of each duplicate gene pair with the chosen *E*_div_, and the second table provides counts of classifications obtained with each of five *E*_div_ values. Figure 1 displays example output figures generated by application of *CDROM* to spatial gene expression data of duplicate genes that arose after human-chicken divergence (from [3]). In Fig. 1a, we applied the default method, in which we did not specify parent and child copies. Thus, duplicate gene copies are labeled as D1 (duplicate 1) and D2 (duplicate 2) in the *CDROM* output files. The resulting output figure depicts a single combined distribution for *E*_D1,A_ and *E*_D2,A_. In Fig. 1b, we specified parent and child copies and, thus, the output figure displays separate distributions for *E*_P,A_ and *E*_C,A_.

Both output figures in Fig. 1 suggest that most pairs of duplicate genes are retained by conservation, consistent with the findings of Assis and Bachtrog [3]. However, in Fig. 1a, the rightward shift in the distribution of *E*_D1,A_ + *E*_D2,A_ indicates that a small proportion of duplicate genes have diverged in expression from their ancestral genes. In Fig. 1b, *E*_C,A_ is shifted to the right, but *E*_P,A_ is not, suggesting that expression divergence generally occurs in child, and not parent, copies. Thus, specifying parent and child copies is advantageous because it can help the user pinpoint which duplicate gene copies have acquired new expression profiles, and potentially have evolved novel biological functions as well.

## Conclusions

Though gene duplication is thought to play a central role in the evolution of novel phenotypes, the mechanisms driving the functional evolution of duplicate genes remain unclear in most species. Assis and Bachtrog [2] recently developed the first approach for classifying these mechanisms by comparing gene expression profiles of duplicate genes in one species to those of their ancestral single-copy genes in a sister species. *CDROM* implements this phylogenetic approach in an easy-to-use and flexible R package, making it accessible to all researchers and applicable to any organisms in which gene expression or other quantitative trait data are available. Thus, researchers can apply *CDROM* to expression data from a variety of species, leading to an enrichment in our understanding of general principles about the origins of phenotypic novelty and complexity.

### Ethics approval and consent to participate

Not applicable.

### Consent for publication

Not applicable.

### Availability of data and materials

The *CDROM* R package, source code, and user manual for running the R package are freely available to download from CRAN at https://cran.rstudio.com/web/packages/CDROM/. Additionally, the *CDROM* R source code, user manual for running *CDROM* from the source code, and sample dataset used to generate Fig. 1 in this manuscript can be accessed at www.personal.psu.edu/rua15/software.html. The only requirement for running *CDROM* is installation of the R software environment.
